# Being in-between and lost in the discharge process—An excursus of two empirical studies of older persons’, their relatives’, and care professionals’ experience

**DOI:** 10.3402/qhw.v7i0.19678

**Published:** 2012-11-06

**Authors:** Ingbritt Rydeman, Lena Törnkvist, Lars Agreus, Karin Dahlberg

**Affiliations:** 1 Department of Neurobiology, Care Science and Society, Centre for Family Medicine, Karolinska Institutet, Huddinge, Stockholm, Sweden; 2 Linnaeus University, Department of Caring Science, Växjö, Sweden

**Keywords:** Discharge process, lifeworld, older person, relative, caring practice

## Abstract

The discharge process (DP) is full of well-known risks, and a comprehensive and well-executed DP is especially important for older people with multiple health problems and continuing care needs, as well as for their relatives. Few studies focus on the experiences with the DP by older people in need of home care nursing and their relatives. Therefore, the aim was to deepen the understanding of the DP as a phenomenon described by older people, their relatives, and care professionals. The method is an excursus of the findings of two previously published research studies. By using the Reflective Lifeworld Research approach, the empirical findings were further interpreted with lifeworld theory. The results describe the essential meaning of the phenomenon of DP in relation to healthcare needs. The illness and the DP can be viewed as a course of action where the familiar becomes unfamiliar for older people and their relatives, entailing an insecure future existence characterized by the experience of being *in-between*. The DP is marked by bodily and existential needs. The older persons and their relatives are lost in the hospital context and trying to influence life and adapt to life circumstances, while being relentlessly dependent on care professionals. Care professionals work from both an organizational and a medical approach. Disharmony and disagreement seem to arise easily among the professionals regarding the planning negatively affecting the patients and their relatives.

More efforts are needed in the DP to empower older people and their relatives to go on with their life at home. The caring practice needs to more clearly meet and address the individual needs of older people and their relatives and their understanding of their illness. It needs to give them lifeworld and life goals to alleviate their suffering and to help them adjust to their new situation.

A challenging issue in many countries is to improve and ensure the continuity and safety of care in the discharge process (DP). This process can be described as a set of actions by hospital professionals to guarantee that patients’ transition from hospital to home meets their continued need for follow-up by primary healthcare and/or social service workers (Coleman, Mahoney, & Parry, 2005; SOSFS, 2005). The DP is especially important for older persons with multiple health problems and needs as well as for their relatives (Shepperd et al., 2010). Well-known deficiencies include lack of patient-centredness (Efraimsson, Sandman, Hyden, & Rasmussen, 2004), patient participation, and co-operation among care providers involved in older patients’ discharge from hospital (Bull, 2000; Fairhurst et al., 1996; Forster, Murff, Peterson, Gandhi, & Bates, 2003; Mistiaen, Duijnhouwer, Wijkel, de Bont, & Veeger, 1997). Information and knowledge about medicine following discharge is reported to be lacking for many older persons (Driscoll, 2000; Graham, Ivey, & Neuhauser, 2009; Knight, Thompson, Mathie, & Dickinson, 2011). Moreover, inappropriate medication treatment is a well-documented reason for hospital admission (Hanlon et al., 2006; Rytter et al., 2010). Any deficiencies in the DP may lead to unnecessary suffering for the patients and their relatives (Bull, 2000). It may also jeopardize the patients’ ability to manage and to receive appropriate support at home and may lead to high costs for society due to readmission to hospital and unplanned acute measures (Rydeman & Tornkvist, 2006a; Jencks, Williams, & Coleman, 2009; Luker, Wilson, Pateman, & Beaver, 2003).

A growing body of knowledge suggests that persons who receive frequent care from different providers are more vulnerable for breakdowns and hospitalizations and thus need comprehensive DP (Forster et al., 2003; Naylor & Keating, 2008). Older patients are often already in an exposed position due to their need of care. The DP is therefore an urgent matter for them and society. A literature review highlights a lacks of studies on the older patients’ perspectives of the DP and their overall matters (Foss & Askautrud, 2010). Few studies describe the experiences of the DP among older persons in detail, and there is lack of knowledge that goes beyond common explanations. However, some studies show that older persons prefer and want to participate in the DP (Foss & Askautrud, 2010; Huber & McClelland, 2003). Moreover, studies focusing on organizational, professionals, and family carers’ perspective are more common. No studies of older persons in need of home care nursing and the DP were found, and in particular, we found no studies using a lifeworld approach.

The aim of this study was to describe experiences with the DP among older persons, their relatives, and care professionals. The aim was also to deepen the understanding of the phenomenon described by previous empirical studies.

## Method

This article is based on an excursus of previously published research (Rydeman & Tornkvist, 2006b, 2010). The aim of the original empirical project was to explore the DP at the hospital for older persons in need of home care nursing. By using Reflective Lifeworld Research, we returned to the empirical findings of the project, which were further interpreted with lifeworld theory (Dahlberg, Dahlberg, & Nyström, 2008). The two studies we build on include the older persons, their relatives, and care professionals involved in DP. The studies completed each other in a useful way for this excursus.

In the first study, we held eight focus groups and interviewed care professionals (*n*=31) with DP experience (Rydeman & Tornkvist, 2006b). The participants were nurses from inpatient care, outpatient care, and municipal care and social workers from social services. The aim of the study was to understand the different professionals’ DP experiences using a phenomenological method (Malterud, 1998). Focusing on older patients’ perspective revealed three themes: *framework*, *basic values*, and *patient resources*, which were important for care professionals’ co-operation, actions, and the outcome of the DP. The overall structure comprised the patients’ vulnerability, dependence, and exposed situation in the DP. Different factors within the themes were associated with various difficulties and problems for the care professionals and influenced the patients’ chances of having an optimal DP.

In the second study, patients aged 65 and older (*n*=14) receiving regular home care nursing, and their relatives (*n*=12) were interviewed about their experience with the DP (Rydeman & Tornkvist, 2010). The aim was to examine their experience with the DP and to highlight aspects of importance for perceived quality. Grounded theory (Glaser, 1979; Glaser & Strauss, 1967) was used revealing two main categories: *preparation areas* and *preparation skills*, which determined whether the older persons and their relatives felt prepared or unprepared for life at home at the time of discharge. Feeling prepared meant having a satisfactory concept of how life at home would be. The *preparation areas* included caring issues, activities of daily living, and where to turn to in case of foreseen or unforeseen needs. The *preparation skills* among professionals included caring competence, individualized commitment, and planning for life at home, all of which are important to satisfy the needs of the older persons and their relatives in the *preparation areas*. When the professionals had adequate *preparation skills*, that is, a guiding approach, the individual needs of the patients and/or their relatives in the DP were satisfied. In contrast, when the care was provided from an unstructured approach, it obstructed them to feel prepared for life at home. The own approach of the older persons and their relatives was also important in satisfying needs.

The comprehensive understanding from the studies showed that the older persons and their relatives as well as the professionals at the hospital viewed the DP as ambiguous. The care and planning were described as fragmented. Whether the presentation and performance of the DP was a fact or planned over a time span, caregivers from outpatient care and social services only played a minor role in the DP despite the fact that they are expected to take over responsibility for care.

The empirical results raised new questions that induced the excurses: there seems to be both correspondence and incongruity from the perspectives of older persons, their relatives, and care professionals. What are the meanings of this? What does the DP and caring practice in this context mean for older persons on a deeper level of understanding?

### Methodological framework and its practise

The excursive interpretation is based on reflective lifeworld research and the analytic principles described by Dahlberg et al. (2008). Lifeworld-based research is focused on the world as it is experienced prior to the formulation of any hypothesis in order to explain it. The process of the analysis can be described as movement between whole–parts–whole (Dahlberg et al., 2008).

Based on the interview data and the results from two empirical studies, the focus of the excursion was to deepen and expand the understanding of the phenomenon, the DP. No new data were added. The text units behind the themes (Study 1), categories (Study 2), as well as the results were re-read to get a sense of the whole. In this new analysis, further meanings were displayed. In the common results of the empirical studies, the patients and their relatives articulated that they are “on their way home but not yet there” and that they are “at the hospital but have not yet left”. With the phenomenological philosophy as a framework, these experiences lead us to an understanding of “*the in-between*”. As another example of the excursive analysis, we noticed that the meaning of vulnerability was not explicit in Study 2. However, on re-reading the data with a lifeworld perspective, we found vulnerability implicitly described as how carers were making decisions “over their heads”. The experience of vulnerability could also be seen in expressions of their frailty and worries of how it will be at home.

As in other phenomenological interpretive analysis, focus was upon meanings and how they are related to each other, that is, forming patterns of meaning. As a result of the excursive analysis, an essential meaning of the phenomenon was described followed by its three constituents. These represent different aspects of the phenomenon, and together they comprise the phenomenon as a whole. All meanings are understood as contextual.

All scientific research and not least an excursive understanding of a phenomenon requires a capacity of researching openness in understanding the phenomenon on its own premises, which is called “bridling” (Dahlberg et al., 2008). The researcher adopts a reflective stance to prevailing assumptions and theories, as well as to the previously described findings, so that the immediate, spontaneous, or taken-for-granted understanding does not affect the analysis in an uncontrolled way. By bridling the process of understanding, the opportunity for seeing something new is strengthened.

### Theoretical framework

The excursus is based on lifeworld theory, which was originally described by phenomenological inspired philosophers in southern Europe. Husserl (1970/1936) is understood to be the founder of the phenomenological movement, from which the lifeworld (Lebenswelt) became an important idea in European philosophy. Merleau-Ponty (1995/1945), Heidegger (1998/1927) and Gadamer (1995/1960) took up the thread and describe the same or similar existential themes. The lifeworld theory has ontological as well as epistemological and methodological implications, and in an essential way it characterizes humans’ existence, our attitude, and experience of everything else that we claim to be our world.

Understanding people and their lives in terms of their lifeworlds constitutes the way with which we understand the world. The lifeworld is thus the actual basis and starting point for the everyday world and for a person's understanding. According to Husserl (1970/1936), the lifeworld is also the starting point for scientific work. The lifeworld is the lived world, the world that is “given” in all experience, thought, and action. Understanding the lifeworld as the world of experience helps us to understand, explain, and describe in a basic way both the everyday world in general and when it is characterized by health, suffering, and caring. Simultaneously, the lifeworld is an individual world and a shared world. We live together and interact with others and these relationships influence the subjects’ being in the world. For example, patients are essentially affected by their relationships with care professionals and relatives (Bengtsson, 2005; Dahlberg et al., 2008; Dahlberg, Todres, & Galvin, 2009; Todres, Galvin, & Dahlberg, 2007).

The lifeworld is an imminent and transcendent world. A person's lifeworld does not have its own existence. It is fundamentally interwoven with and cannot be separated from the person who is described, and is difficult to discern with one's senses. It is characterized both by temporality and by spatiality: the lifeworld is essentially “here and now,” but it also involves the movement between the present, past, and future, and the different spatial horizons move with the lived body (Merleau-Ponty, 1995/1945).

A central part of the phenomenological philosophy and the lifeworld idea is intentionality. First, intentionality concerns how everything that we think, feel, and do in relation to the world around us has its starting point in how this world is experienced by us, and how we observe things in the world around us (Cavalcante Schuback, 2006). Second, it is made clear how we, at a basic level, always experience the world as something; that is, how everything we see, hear, or in other ways perceive always has significance, a meaning for us (Dahlberg et al., 2008).

### Ethical consideration

All ethical considerations required were met, and the both studies have ethical approval from Ethics Committee of Karolinska Institute at Huddinge University Hospital.

## Results

For the older person and their relatives, hospitalization and DP are understood as a critical event with the illness making the meaning of life's fragility abruptly explicit and an unpredictable threat to getting on with one's life and its minor and major life projects. The illness and hospitalization disturb life, but nevertheless there is a strong wish to come home again. This is the context in which the DP operates.

The DP should be a health-promoting bridge between the hospital and the older persons’ home and support their intentions to go on with their lives. Our research results reveal that this bridge can be absent or weak and that the DP comprises experiences of being seen and not seen, equality and inequality, inclusion and alienation, and threat as well as safety.

The DP is a phenomenon that is characterized by experiences of being *in-between*. The older persons have to relate to *in-between* experiences that are *contextual*, *bodily*, and *existential*. *The contextual in-between* implies being part of a hospital context that the older persons and their relatives are more or less familiar with and do not fully understand or control. It is a context that they are being controlled and one in which they have to rely on others’ expertise. The *bodily in-between* includes the impact of illness and ageing on body and bodily fragility with aspects such as limited mobility and loss of abilities influencing one's self-perception and independency. The *existential in-between* includes being faced with altered life premises including losses and death, and the realization of life goals one strives to fulfil. The relationship with care professional and others, bodily conditions and life circumstances influence the *in-between* experience.

The DP is marked of bodily and existential diversity intertwined with bodily and existential needs. A decisive factor for how the DP is going to proceed is how the care professionals are able to handle the discharge situation. The caring practice builds upon encounters, relations, perceptiveness, and openness toward the patients’ and their relatives’ needs and awareness of their vulnerability. When carers treat older persons and their relatives with respect for their experiences and life coherence, they feel heard, seen, and included, and as a result, feelings of trust and safety increase. Without the professional support and lack of co-operation among them in the DP, the older persons run the risk of being lost and exposed in the DP.

To further display the meaning of the care and the experiences of *in-between*, the results of the excursus are further described in the form of meaning constituents, which reflect the DP's, more contextual and meaning variation. The three constituents of the experience of the DP as *being in-between* are not completely separated but intertwined: vulnerability—lost in the life context, influencing one's life, and adapting to life circumstances.

### Vulnerability—lost in the life context

Contextual, bodily, and existential in-between experiences are displayed as vulnerability, which relates to the context of being at hospital but on their way home, to the bodily frailty of not being ill but not well enough to manage a life at home and consequently, to a shaky existence. Being in hospital means being in an unfamiliar context that lacks existential coherence excluded from the familiar ones and in need of help. Patients want their suffering and needs to be understood. Due to their often-complex health situation and age, it can be difficult to formulate what they need, but they expect the care professionals to know, meet, and discuss this with them. If they instead encounter a one-sided understanding, focusing on biomedical aspects, and they are being treated as a group, that is, elders or objects, they develop feelings of loneliness, vulnerability, and alienation. Their life coherence is brushed aside by impersonal care and ruled and delimited by care professionals’ pre-understanding and medical view. The older persons and their relatives need care that listens and affirms their lifeworld and how they perceive the present situation. Such care would alleviate and reduce their vulnerability. The lack of such care leaves the older persons and their relatives alone and further attaches them to the *in-between* experience.

When there is no true will to reach the older persons or their relatives, their experience is an absence of care and unfair treatment. Despite the unequal relationship with the care professionals, the older persons and relatives are dependent of and rely on their support—they have no other choice. When the professionals determine the DP plan without listening to the older persons and their relatives, they feel diminished and hard to influence their situation. Uneasy feelings are created, intruding one's life. To deal with this vulnerability, the older persons and their relatives try to hide their feelings by ignoring the power of the professionals, by arguing for their own cases, or by conforming to the circumstances, withdrawing or relied on one's relatives.

The care professionals perceived the older persons as alone and in a disadvantageous position in the DP and that their illness threatened their desired life situation. However, they also described a disharmony among the caregivers about how they emphasized different aspects and how they make different assessments and plans of the older persons’ care. Even if the professionals want to do well and take responsibility for the older persons’ care, they cannot control the DP rules and its attendant medical approach. Furthermore, ambiguous responsibilities among caregivers can cause unforeseen problems, and patients may fall through the cracks. Organizational circumstances and obscure structures result in disagreement, frustration, and complications in co-operation.

### Influencing one's life

Older persons’ self-determination seems to be highly connected to their relationship with the care professionals at the hospital and in relation to the DP. It is important for the older persons to understand the new life situation and also to influence their everyday life. Here, the experience of existential *in-between* is apparent.

Being at the hospital but on their way home, they expect the care professionals to include them in any new circumstances and in care planning, and not force them into something. The older persons and their relatives reported that they felt safe, supported, and welcome to ask questions, when the care professionals explained the caring process, treatment, and planning. However, when the care professionals’ agenda is ruled by a biomedical approach, that is, illness oriented, the older persons’ individual needs to participate in the health and care processes are not met. They reported feeling stress when excluded from the planning or when they felt that their entire and complex situation was not seen or taken care of. They fight for their influence on the concerns of everyday life, but often lose their freedom to make any choices. Consequently, their sense of coherence is lost. Collisions occurs when these different perspectives meet and the older persons’ and their relatives’ abilities to live authentically decrease.

To try to protect themselves, the older persons and their relatives must adapt to prevailing hospital structures and believe in the professionals’ good will. The older persons reported how they hoped the situation would improve when they returned home where they could regain self-determination. If they had relatives to rely on, mainly the spouses, they would ask them to help them and participate in the homecoming planning.

Even the professionals described how they sensed the challenge for the patients and their relatives to influence or question the care planning. They also saw that autonomous decisions from the patients are not always welcomed. For example, the older persons sometimes refuse social services at home, which the professionals deem necessary. The older persons can also find themselves in the middle of disagreements between professionals and relatives, or among relatives, and be persuaded into care alternatives that they do not want.

### Adapting to life circumstances

The older persons and their relatives strive to adjust to illness, the health care, and the care professionals’ approach and to regain balance in a fragile existence and life circumstance. They hold on to the important independence and try to create meaning-coherence under actual circumstance. Living with worsening health, repeated hospitalizations, and a fragile body is a reminder of existential fragility, as well as an existential *in-between* situation. When illness causes major changes in life, a crisis-like experience unfolds. In such situations, it is important that professionals are present and taking responsibility, which helps contribute to alleviation. Understanding the illness and reasons for the suffering can be a relief. However, a situation when the older persons’ needs are not acknowledged or addressed at hospital induces insecurity, seriously affecting the DP and homecoming. The homecoming becomes distressing and filled with uncertainty.

In the midst of the DP, the older persons and their relatives do not take everyday life and future for granted, and the *in-between* experience is manifested. They are not staying at the hospital, but they are not at home; they want to return home and carry on with life, but the familiar environment and everyday world is different and could mean that everyday life will not work anymore; they want to live an autonomous life but face dependency; they are the same persons as before, yet they are not. The illness and the care have presented them with an undefined and ambiguous situation.

Professionals said that they appreciate it was hard for the older persons and their relatives to decide prior to the discharge what kind of support they needed at home. At the same time, they reported how hard it was for them to give the right support.

## Discussion

The illness, the associated hospitalization, and the DP make the terms of life and its fragility abruptly explicit for the older persons. Their *in-between* existence is evident as well as difficult: they are supposed to be well enough to go back home, but they still feel ill; they are supposed to make decisions about life at home, but still being at the hospital, they are ruled by the carers and the hospital regime; the lived body must now fulfil all demands from life at home, yet they feel weakness, which fills them with anxiety and worry.

Illness specifically signifies the altered access to a good life through a healthy body and the vulnerability in this relationship. Everyday life can no longer be taken for granted, and the older persons and their relatives feel lost and misplaced under the altered life circumstance. The illness and the DP can be understood as events in which the familiar becomes unfamiliar, which entail an insecure presence and future existence. Control over one's life is jeopardized.

The DP as a phenomenon is characterized by movements of belonging and alienation, which becomes apparent in the older persons’ descriptions of how the care proceeded from an objective and one-sided understanding of them. When the older persons are reduced to non-persons, to a “diagnosis” or an “object” that will leave hospital and go home, they lose their power to create something good from the in-between situation. All *in-between* situations include possibilities as well as risks, but to benefit from them, the older persons need to be personally acknowledged for their potentials and supported to re-orientate.

### Vulnerability—lost in the life context

Besides the illness, the hospital context and performance of the DP also conduce to vulnerability and uncertainty for the older persons and their relatives. As reported in several studies, vulnerability is obvious at hospital and in care due to the dominance of the medical model of care and inflexible dehumanizing care (Connolly et al., 2009; Summer-Meranius, 2010). Patients are always more or less exposed and vulnerable due to their suffering and need of care (Dahlberg & Segesten, 2010).

The older persons felt the care professionals viewed them as objects, as home-goers, and from the group of “elderly” rather than individuals. As a result, they lose their subjectivity, which is described as hurting and demanding as well as insulting. Toombs (1993) describes experiences among patients that are “objects of investigation”. Our findings illustrate her theoretical descriptions of how illness transforms the known into the unknown. Also Nystrom, Dahlberg and Carlsson (2003) illustrates how distanced care professionals proceeding from pre-determined action plans contribute to diminished room for individual encounters and to situations where patients’ individual needs are not met. In care provided from routines and “the ways we always do things,” the lifeworld is less likely to be heard; it is shouted down by the strong medical voice (Dahlberg & Segesten, 2010; Efraimsson, Sandman, & Rasmussen, 2006).

Also, ageism is reported to be a base for preconceptions and discrimination against older persons and the most accepted existing kind of pre-conception today (Andersson, 2008). The unequal relation perceived by the older persons at hospital as well as the illness accentuates vulnerability and loss of control. Despite these well-known facts, the health care system in many European countries is still reported to fail older persons who are among the most vulnerable (Genet et al., 2011; Socialstyrelsen, 2011; Tingle, 2012).

### Influencing one's life

The older persons and their relatives described dependency and having to rely on care professionals to involve and guide them at hospital. They talked about how they needed to orientate in the unfamiliar situation in order to go on with minor and major life projects. A strong will to influence everyday life concerns and to live an autonomous life is obvious in our data, but the consequence is that self-determination seems to decrease and dependency increase at hospital and in the DP. In Svenaeus’ (2003) analysis of meaning of illness, the ill persons may face helplessness to varying extent, but in order for health to be dominant, they should still be in charge of their lives. The procedures at hospital and the way staff relate to older patients and their relatives, their way of respecting or disrespecting the patients and their lifeworld can be a support for or continuous threat to their independence and autonomy.

Independence is cited by older persons as particularly important for retaining dignity at hospital (Webster & Bryan, 2009). Older persons in care need freedom to make their own choices and plans for everyday life at home despite an uncertain situation. Older persons with multi-morbidity in the Summer-Meranius study (2010) described their home as the place where they could be themselves and independent despite needing help. Independence means to make autonomous decisions and participate in decisions regarding everyday life, which correspond with the older persons’ desires in our study.

There are alternatives to the prevailing caring practice. Dahlberg et al. (2009) suggest lifeworld-led care where care professionals offer not only technical solutions but also knowledge that understands both freedom and vulnerability of people's journeys as they struggle with different health-related conditions. Professionals need to acknowledge the existential impact but also patients’ expertise in order to lead the care from an expanded view of knowledge. Gustafsson (2004) and Porn (1993) describe a view of holistic care highlighting the persons’ individual circumstances, for example, their life plan, and respecting the persons’ goals. Furthermore, Gustafsson (2004) also stress the need of confirming encounters.

### Adapting to life circumstances

The illness and the caring practice present the older persons and their relatives with an uncertain situation to which they must adapt. The individual reaction to illness and care has particular relevance for the life situation and constitutes the specific meaning that both illness and care have for life. The older persons struggle to find ways to go on with life and fragile bodies. Despite diseases and illness, one can realize projects and perceive well-being through, for example, self-formulation and creative ways of relating to illness and gaining control of the situation again (Mayan, Morse, & Eldershaw, 2006).

In our study, the older persons reported that their relatives could be of great help but other support might be needed. Many older persons are reported to encounter a variety of problems within the first week after being discharged from hospital (Mistiaen, Francke, & Poot, 2007). Frail older persons benefit from individualized follow-up after being discharged from hospital as suggested and tested in the USA (Naylor, 2012). By monitoring how it works and keeping a lifeworld approach in mind, professionals can invite the older persons and their relatives to discuss their experiences and concerns related to their illness, the hospitalization, everyday life and their minor as well as major life projects, their sense of vitality, life lust and whether they have enough movement and/or stillness in their lives (Dahlberg & Segesten, 2010). Outpatient care and social services could offer follow-ups, and further support could easily be initiated, for example, training, assisted device, and clarification of medical treatment.

### Methodological issues

The excursus using reflective lifeworld approach of previous empirical studies contributed to a more profound understanding of the DP going beyond common well-documented explanations. Despite the fact that interview data were collected and analysed according to different qualitative approaches, the studies complemented each other in a useful way and deepened the understanding of the previous findings. Researchers are always challenged of prevailing understanding and of not conceptualizing too early and thus losing the meaning. We found that a reflective stance and the practise of “bridling” increased openness and an opportunity to see something new in the texts. Moreover, we also included a colleague who had not participated in the included studies, which also contributed to methodological rigour and more valid results.

## Conclusion and implications for practice

The DP is full of well-known risks, and more efforts are needed to empower the older persons and their relatives to get on with their life at home. Instead of being a strong bridge between the hospital and home, the DP can weaken the older persons’ life power and hinder their reconciliation. The older persons and their relatives can easily be lost in an *in-between* experience in which their existence is threatened. They are faced with the unfamiliar and unknown in their lifes contexts. Health care professionals meet people in illness when they are most vulnerable. The caring practice can support and strengthen well-being, but when the older persons are not seen as individuals, not invited into the care processes, the care may contribute to the experience of an unfamiliar situation and cause suffering.

We advocate an alternative caring practice, which acknowledges the individual lifeworld and the older persons’ life context. The older persons and their relatives need genuine encounters with engaged and interested care professionals. Such approach means focusing upon the older persons’ and their relatives’ individual situation, their meaning of health and illness, as well as their life goals and other existential aspects. Lifeworld-led care can alleviate their suffering and help them to reconcile with the new situation and find ways to go on with their life projects.
